# Carbohydrate Metabolism Affects Macrophage-Mediated Killing of Enterococcus faecalis

**DOI:** 10.1128/mSystems.00434-21

**Published:** 2021-09-07

**Authors:** Lifan Wei, Feng Xia, Jia Wang, Shujun Ran, Yakun Liang, Wei Zhou, Zhengwei Huang, Jingping Liang

**Affiliations:** a Department of Endodontics and Operative Dentistry, Ninth People’s Hospital, College of Stomatology, Shanghai Jiao Tong University School of Medicine, Shanghai, China; b National Clinical Research Center for Oral Diseases, Shanghai, China; c Shanghai Key Laboratory of Stomatology & Shanghai Research Institute of Stomatology, Shanghai, China; d Shanghai Institute of Precision Medicine, Ninth People’s Hospital, Shanghai Jiao Tong University School of Medicine, Shanghai, China; e State Key Laboratory of Bioreactor Engineering, East China University of Science and Technology, Shanghai, China; Chan Zuckerberg Biohub

**Keywords:** *Enterococcus faecalis*, transposon insertion sequencing, carbohydrate metabolism, macrophage-mediated killing

## Abstract

Enterococcus faecalis, an opportunistic pathogen that causes severe community-acquired and nosocomial infections, has been reported to resist phagocyte-mediated killing, which enables its long-term survival in the host. Metabolism, especially carbohydrate metabolism, plays a key role in the battle between pathogens and hosts. However, the function of carbohydrate metabolism in the long-term survival of E. faecalis in phagocytes has rarely been reported. In this study, we utilized transposon insertion sequencing (TIS) to investigate the function of carbohydrate metabolism during the survival of E. faecalis in RAW264.7 cells. The TIS results showed that the fitness of carbohydrate metabolism-related mutants, especially those associated with fructose and mannose metabolism, were significantly enhanced, suggesting that the attenuation of carbohydrate metabolism promotes the survival of E. faecalis in macrophages. The results of our investigation indicated that macrophages responded to carbohydrate metabolism of E. faecalis and polarized to M1 macrophages to increase nitric oxide (NO) production, leading to the enhancement of macrophage-mediated killing to E. faecalis. Meanwhile, E. faecalis automatically decreased carbohydrate metabolism to escape from the immune clearance of macrophages during intracellular survival. The shift of primary carbon resources for macrophages affected the ability to clear intracellular E. faecalis. In summary, the results of the present study demonstrated that carbohydrate metabolism affects the macrophage-mediated killing of E. faecalis.

**IMPORTANCE** E. faecalis has become a major pathogen leading to a variety of infections around the world. The metabolic interaction between E. faecalis and its host is important during infection but is rarely investigated. We used transposon insertion sequencing coupled with transcriptome sequencing to explore the metabolic interaction between E. faecalis and macrophages and uncovered that the shift of carbohydrate metabolism dramatically affected the inflammatory response of macrophages. In addition, E. faecalis attenuated carbohydrate metabolism to avoid the activation of the immune response of macrophages. This study provides new insights for the reason why E. faecalis is capable of long-term survival in macrophages and may facilitate the development of novel strategies to treat infectious diseases.

## INTRODUCTION

Enterococcus faecalis is an opportunistic pathogen that primarily colonizes the oral cavity and intestinal tract (1). Due to the high growth rate and strong adaptability of this bacterium to various conditions, such as *in vivo* environments (2), extreme conditions (3), and the stress of various antibiotics (4), E. faecalis has become a major pathogen leading to a variety of community and nosocomial infections (1). According to clinical reports, E. faecalis is responsible for 7% to 15% of wound infections (5), 5% to 8% of bacteremia (6), 5% to 26% of endocarditis (2), and more than 80% of secondary root canal infections (7). Among the different severe infections caused by E. faecalis, bacteremia and endocarditis are the two most life-threatening infections with a morality rate of more than 17% (2). During infection, innate immunity is first activated to defend against pathogenic invaders (8). Leukocytes, especially macrophages, are recruited to infectious sites to phagocytose and eliminate pathogens (9), which is followed by the delivery of pathogen-associated molecular patterns (PAMPs) from pathogens to activate adaptive immunity to further clear the invading pathogens (9, 10). However, E. faecalis is capable of resisting the phagocyte-mediated killing, delaying the activation of adaptive immunity, promoting its survival inside host cells (11–13), and finally leading to chronic inflammation or systemic infection that is difficult to be cured by antibiotics (2). Therefore, elucidating the pathogenic mechanism by which E. faecalis resists phagocyte-mediated killing is the key to combating E. faecalis-associated infectious diseases.

Metabolic interactions between pathogens and hosts have recently been reported to occur during infection. The host enhances glycolysis for rapid ATP production to trigger an immune response and polarization of macrophages to M1 (14), increases arginine and metabolism for nitric oxide (NO) production to kill bacterial invaders (15), and limits iron acquisition by pathogens (16). On the other hand, pathogens coordinate their own metabolism to adapt to host conditions. For example, Mycobacterium tuberculosis not only decreases primary metabolism and enters a dormant status after successfully colonizing macrophages (17) but also utilizes the energy metabolism of host cells to increase its fitness in host cells (18–20). However, the metabolic interactions between E. faecalis and macrophages have yet to be investigated.

Transposon insertion sequencing (TIS) has been widely used to identify the genetic determinants of bacterial survival or growth under various conditions (21, 22). In the present study, we used TIS to identify the key metabolic genes or pathways affecting E. faecalis survival in macrophages. The results showed that a number of metabolism-related mutants, especially those involved in fructose and mannose metabolism, promoted E. faecalis survival in macrophages. Subsequently, the function of carbohydrate metabolism during E. faecalis survival in macrophages was validated and investigated.

## RESULTS

### E. faecalis resists macrophage-mediated killing.

E. faecalis has been reported to resist macrophage-mediated killing (11, 12). To better characterize macrophage-mediated killing of E. faecalis, the survival dynamics of the E. faecalis strains OG1RF and ATCC 33186 in RAW264.7 macrophages were monitored. Twenty-four hours postinfection (h.p.i.), the survival of Lactobacillus lactis and Escherichia coli in RAW264.7 cells dramatically decreased by 137-fold and 70-fold, respectively (see Fig. S1A in the supplemental material). In contrast, E. faecalis strains OG1RF and ATCC 33186 in RAW264.7 cells survived well, exhibiting a reduction of less than 2-fold (Fig. S1A). At 48 and 72 h.p.i., L. lactis and E. coli were nearly completely cleared, while E. faecalis strains OG1RF and ATCC 33186 maintained a high level of survival in RAW264.7 cells (Fig. S1A), suggesting that E. faecalis resisted macrophage-mediated killing to promote its survival. To observe the survival of bacteria in macrophages, transmission electron microscopy (TEM) was performed, and the results showed that bacteria located in phagosomes after internalization and the bacterial counts of the E. faecalis strains OG1RF and ATCC 33186 in infected cells were significantly higher than those of L. lactis and E. coli in infected cells after 24 h of infection (Fig. S1B), which was in keeping with the results shown in Fig. S1A.

10.1128/mSystems.00434-21.1 FIG S1 E. faecalis resists macrophage-mediated killing. (A) RAW264.7 cells were infected with the E. faecalis strains OG1RF and 33186, Lactobacillus lactis, and E. coli DH5α at an MOI of 10. Cells were washed and further incubated with DMEM supplemented with 10% FBS containing vancomycin and gentamycin to kill extracellular bacteria 2 h.p.i. The number of viable bacteria within host cells was counted at 2, 24, 48, and 72 h postinfection. More than three replicates were performed. (B) Characterization of bacterium-containing phagosomes by TEM. RAW264.7 cells were infected with the above strains at an MOI of 10. After 24 h of infection, cells were fixed and processed for imaging by transmission electron microscopy. Representative images are shown. Download FIG S1, TIF file, 2.6 MB. Copyright © 2021 Wei et al. 2021 Wei et al. https://creativecommons.org/licenses/by/4.0/ This content is distributed under the terms of the Creative Commons Attribution 4.0 International license.

### A comprehensive analysis of metabolism-related genetic determinants of E. faecalis resistance to macrophage-mediated killing.

**FIG 1:**
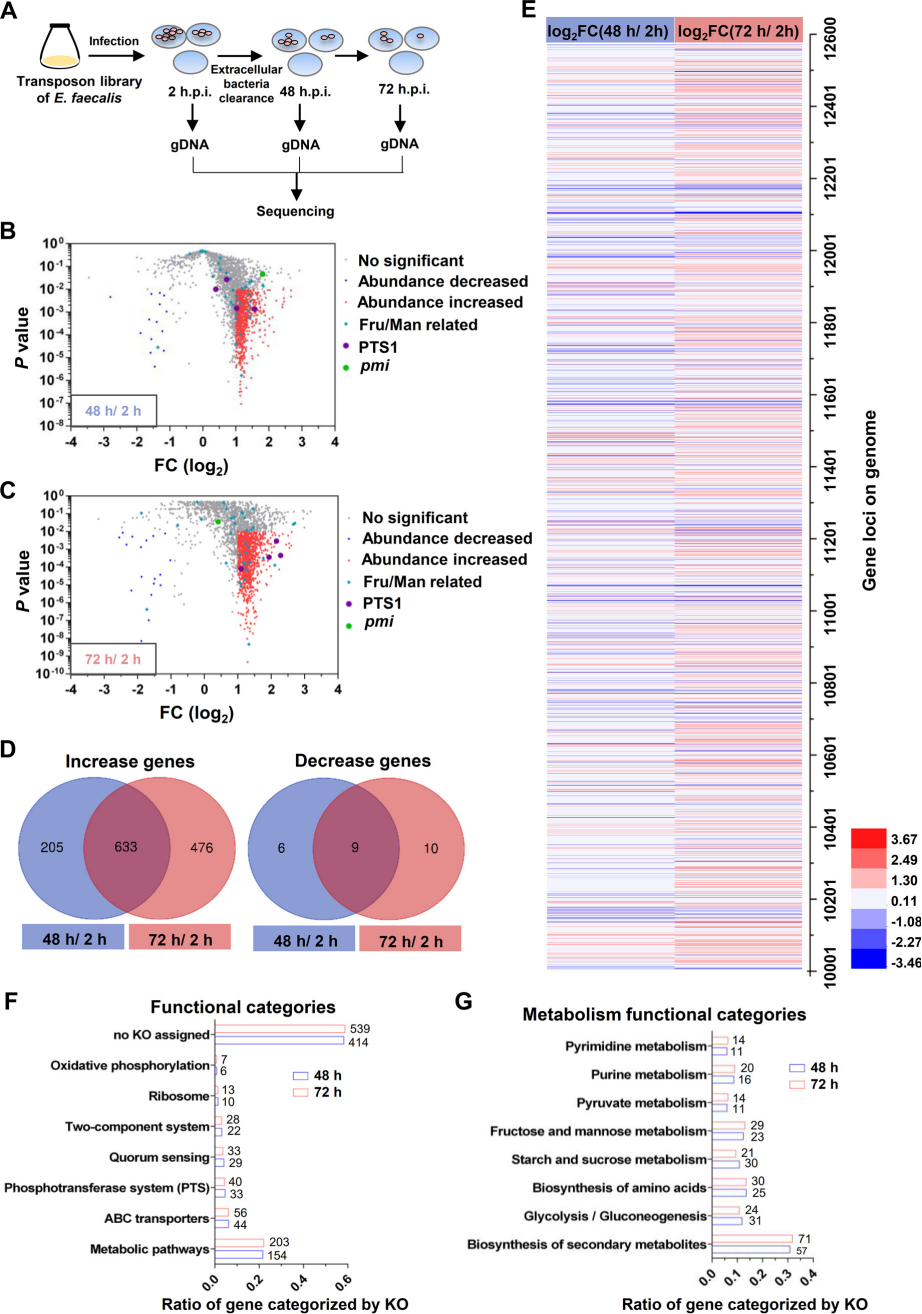
Fitness screening for E. faecalis survival in macrophages by transposon insertion sequencing (TIS). (A) Schematic diagram of genetic determinant screening. Intracellular bacteria were harvested at 2, 48, and 72 h postinfection (h.p.i.), followed by DNA extraction for sequencing. (B and C) Scatterplots of the fold change (log_2_ output/input) and corresponding *P* values of mutants after survival within RAW264.7 cells for 48 h (B) or 72 h (C). The blue and red dots represent mutants with survival decreased (*P < *0.01; log_2_ FC, ≤−1.0) and survival increased (*P < *0.01; log_2_ FC, ≥1.0), respectively. The blue triangles represent fructose and mannose metabolism-related mutants. The purple and green dots represent PTS1 and *pmi*, respectively. Three or two independent biological replicates were performed. (D) Venn diagram depicting the genes with significant differences after 48 or 72 h of infection. (E) Heatmap of the genes with abundances decreased or increased relative to the input (2 h.p.i.). (F and G) KEGG orthology classification of genes with significant differences in fitness. The percentages shown are the fraction of the total number of depleted genes represented by the number of depleted genes/category. The number of depleted genes in each category is shown.

To comprehensively assess the metabolic determinants enabling E. faecalis to resist macrophage-mediated killing, transposon insertion sequencing (TIS) was performed using the transposon insertion mutant library of E. faecalis OG1RF. A schematic diagram of the TIS pipeline employed to identify E. faecalis genetic determinants of resistance to macrophage-mediated killing is shown in Fig. 1A. After RAW264.7 cells were colonized for 2, 48, and 72 h, the transposon insertion mutants were harvested and inoculated into brain heart infusion (BHI) medium for a short incubation period (approximately 2 h) to reduce interference from dead bacteria; this step was followed by genomic DNA (gDNA) extraction and next-generation sequencing (Fig. 1A). The replicates showed a high correlation (see Fig. S2A in the supplemental material). Analysis of the sequencing results by AL-Artemis (23) showed that the transposon mutant library contained 20,852 different mutants (see Table S1 in the supplemental material). Compared with internalized mutants (2 h.p.i.), 853 mutants exhibited a significant difference in abundance (log_2_ fold change [FC] of ≥1.0 or ≤−1.0; *P < *0.01) after 48 h of survival in RAW264.7 cells, including 838 and 15 mutants with abundance increases and decreases, respectively (Fig. 1B and Table S1). After 72 h of survival in RAW264.7 cells, 1,128 mutants showed a significant difference in abundance (log_2_ fold change of ≥1.0 or ≤−1.0; *P < *0.01), including 1,109 and 19 mutants with abundance increases and decreases, respectively (Fig. 1C and Table S1). For the mutants with significant differences, 9 and 633 mutants with abundance decreases or increases, respectively, were enriched in both the 48-h and 72-h groups (Fig. 1D). A heatmap was also generated to display the abundance change for the mutants in the library, and many mutants in the 48-h group showed a similar change as those in the 72-h group (Fig. 1E).

10.1128/mSystems.00434-21.2 FIG S2 (A) Correlation of experimental replicates from the TIS results. The sequencing data from experimental replicates of input libraries (*n *= 3) and output libraries recovered after survival in RAW264.7 cells for 48 h (*n *= 2) and 72 h (*n *= 3) are shown. (B) Transcriptome profiles depicting the expression of fructose and mannose metabolism-associated genes at log and stationary phases in BHI medium. Three independent biological replicates were performed. Download FIG S2, TIF file, 1.1 MB. Copyright © 2021 Wei et al. 2021 Wei et al. https://creativecommons.org/licenses/by/4.0/ This content is distributed under the terms of the Creative Commons Attribution 4.0 International license.

10.1128/mSystems.00434-21.5 TABLE S1 List of transposon insertion mutant library screened for genetic determinates for E. faecalis survival in RAW264.7 cells. Download Table S1, XLS file, 0.8 MB. Copyright © 2021 Wei et al. 2021 Wei et al. https://creativecommons.org/licenses/by/4.0/ This content is distributed under the terms of the Creative Commons Attribution 4.0 International license.

### Attenuation of carbohydrate metabolism promoting E. faecalis survival in macrophages.

The genes with transposon insertions resulting in significant changes in abundance in the 48-h and 72-h groups were further assessed by KEGG orthology (KO) analysis to determine their functions. A total of 154 and 203 genes in the 48-h and 72-h groups were identified as being involved in metabolic pathways (Fig. 1F), including 147 (95.4%) and 197 (97.0%) genes with abundance increases in the 2 groups, respectively. These results imply that the attenuation of metabolism may strongly contribute to E. faecalis survival in RAW264.7 cells. Furthermore, these metabolic pathways were primarily involved in pathways such as biosynthesis of secondary metabolites, glycolysis/gluconeogenesis, biosynthesis of amino acids, and fructose and mannose metabolism (23 and 29 genes in the 48- and 72-h groups, respectively) (Fig. 1G). Some genes in the fructose and mannose metabolism category also overlapped with genes in the phosphotransferase system (PTS) category, which also affected E. faecalis survival in RAW264.7 cells (Fig. 1F and G). According to KEGG analyses, multiple PTSs are carried by E. faecalis, hinting that PTS may play key roles for E. faecalis survival *in vivo* or/and *in vitro*. In addition, fructose and mannose metabolism have been reported to play important roles during infection (24–26). Therefore, we investigated fructose and mannose metabolism and their implication on the survival of E. faecalis in macrophages.

### PTS1 and Pmi regulate carbohydrate metabolism in E. faecalis.

**FIG 2:**
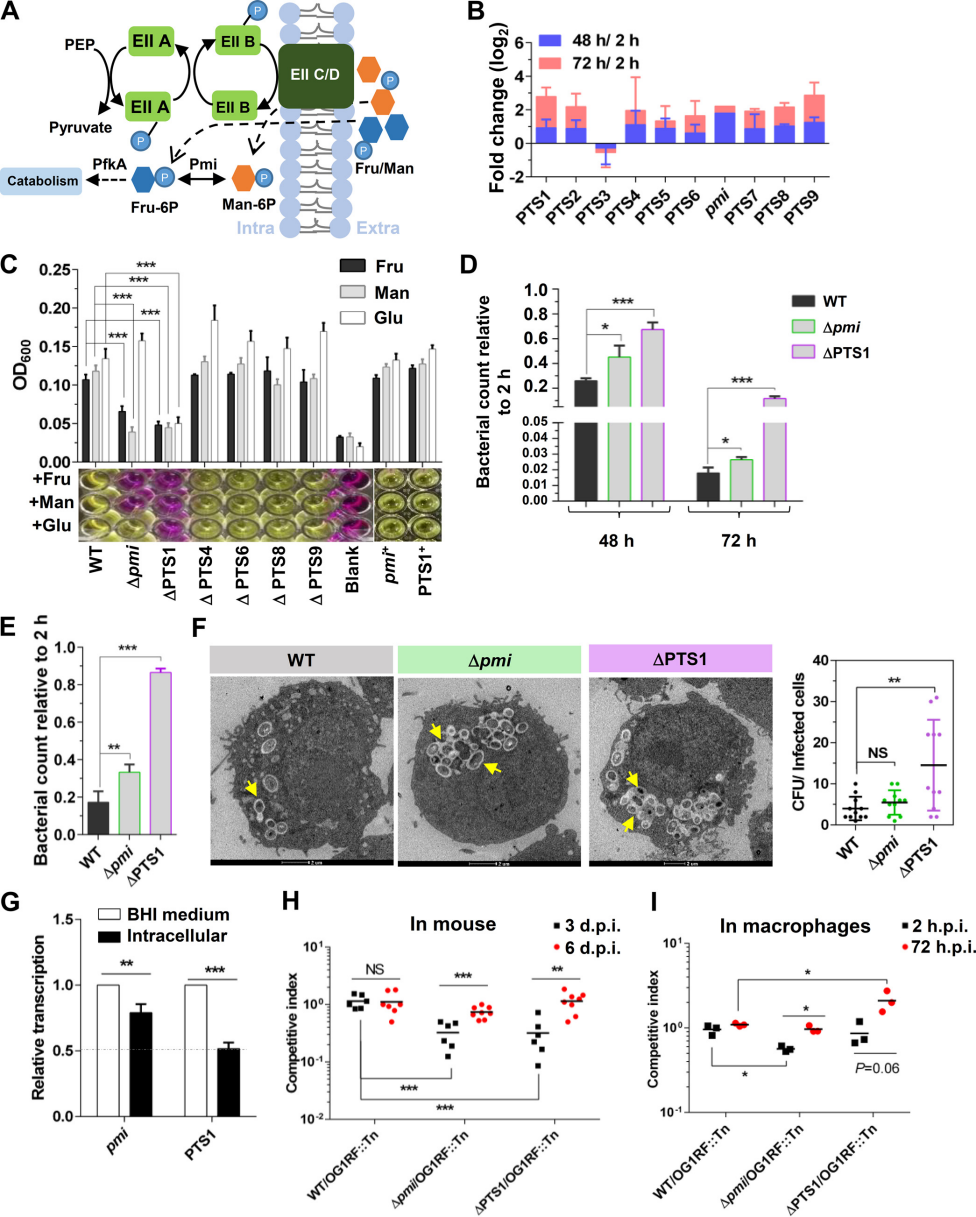
Carbohydrate metabolism affects E. faecalis survival in macrophages. (A) Schematic diagram of fructose (fru) and mannose (man) transport and metabolism in E. faecalis. (B) The fold change (log_2_) of fructose and mannose metabolism-related genes after survival in RAW264.7 cells for 48 h (blue) or 72 h (red). The initial data of this histogram are from the TIS results. (C) Growth of E. faecalis strains in DMEM. Sugar-free DMEM supplemented with fructose, mannose, or glucose was used to cultivate the strains. *n *≥ 3; *, *P* < 0.05; ***, *P* < 0.001 (based on Student’s *t* test). (D and E) The survival of E. faecalis strains in RAW264.7 cells (D) or primary peritoneal macrophages (E) after 48 or 72 h of infection. The viable bacterial number at the indicated time was counted by plating. *n *≥ 3; *, *P < *0.05; ***, *P < *0.001 (based on Student's *t* test). (F) Transmission electron microscopy (TEM) results showing the survival of the E. faecalis WT, Δ*pmi*, and ΔPTS1 strains in RAW264.7 cells at 48 h.p.i. The yellow arrows indicate E. faecalis survival in phagosomes. The bacterial counts in infected cells were calculated. Representative images are shown. NS, no significant difference; **, *P < *0.01 (based on one-way ANOVA). (G) qRT-PCR results showing the expression of PTS1 and *pmi* in RAW264.7 cells. The E. faecalis WT strain was cultured in BHI medium and survived in RAW264.7 cells for 24 h. *n *≥ 3; ***, *P < *0.001 (based on Student’s *t* test). (H andI) The competitive survival of E. faecalis strains in mice (H) and in primary peritoneal macrophages (I). WT, Δ*pmi*, and ΔPTS1 cells were competed 1:1 versus a transposon mutant (insertion in OG1RF_12558 to OG1RF_12559), which was gentamicin resistant and used to distinguish the Δ*pmi* and ΔPTS1 strains. Six or eight mice were used per group. *n *≥ 3; **, *P < *0.01; ***, *P < *0.001; *P* values determined via ANOVA followed by Bonferroni’s multiple-comparison posttest.

Fructose and mannose transport and metabolism in E. faecalis are depicted in Fig. 2A, where the conversion of phosphoenolpyruvate (PEP) to pyruvate releases phosphoric acid, which is gradually transferred to EIIC/D to phosphorylate extracellular fructose or mannose to form fructose-6-phosphate (fru-6P) or mannose-6-P (man-6P) (Fig. 2A) (25, 27). Next, fructose-6-phosphate or mannose-6-phosphate is transferred into cells by EIIC/D, followed by the isomerization of mannose-6-phosphate into fructose-6-phosphate by mannose-6-phosphate isomerase (Pmi) (Fig. 2A). Fructose-6-phosphate is further metabolized by 6-phosphofructokinase (PfkA) to enter glycolysis and the citrate cycle for ATP and secondary metabolite production (Fig. 2A). Notably, E. faecalis carries one copy of *pmi* and nine different kinds of fructose and mannose phosphotransferase systems (PTSs; PTS1 to PTS9), implying that E. faecalis may be strongly dependent on fructose and mannose metabolism to support growth or survival. Most of these fructose and mannose metabolism-related mutants in the library showed different degrees of survival enhancement in RAW264.7 cells after 48 or 72 h of infection, except a PTS3 (OG1RF_10547-10549) mutant, which showed a slight decrease (Fig. 2B).

To further investigate fructose and mannose metabolism in E. faecalis, deletions of these genes were engineered. According to the transcriptional profiles, PTS2 (OG1RF_10340-10342), PTS3, and PTS5 (OG1RF_11549-11552) were expressed only at the log phase in BHI medium (Fig. S2B). In addition, both PTS2 and PTS3 consisted of three genes (Fig. S2B), which may be incomplete for transport function. Thus, the in-frame deletions of PTS1 (OG1RF_10018-10021), PTS4 (OG1RF_11510-11513), PTS6 (OG1RF_11613-11616), PTS7 (OG1RF_12259-12262), PTS8 (OG1RF_12399-12402), PTS9 (OG1RF_12476-12479), and *pmi* were constructed to further investigate the influence of fructose and mannose metabolism on E. faecalis survival in macrophages. All of these deletions were successfully obtained, except for PTS7. According to growth curves, the deletion of *pmi* and PTS1 decreased the growth rate of E. faecalis in BHI medium (see Fig. S3A in the supplemental material). In addition, the ΔPTS1 strain showed a growth defect in Dulbecco’s modified Eagle’s medium (DMEM) supplemented with fructose, mannose, or glucose as the sole carbon resource (Fig. 2C), and the complementation of PTS1 restored the defective phenotype to that of the wild-type (WT) strain (Fig. 2C). The Δ*pmi* strain exhibited markedly reduced growth in sugar-free DMEM supplemented with fructose or mannose as the sole carbon resource (Fig. 2C) but grew normally with glucose as the sole carbon resource. The complementation of *pmi* restored the phenotype to that of the WT strain (Fig. 2C). The E. faecalis ΔPTS4, ΔPTS6, ΔPTS8, and ΔPTS9 strains showed no significant growth defects *in vitro* (Fig. 2C and S3A). These results suggested that PTS1 and Pmi critically regulate carbohydrate (fructose, mannose, and/or glucose) metabolism in E. faecalis.

10.1128/mSystems.00434-21.3 FIG S3 (A) Growth curve of E. faecalis strains in BHI medium. *n *≥ 3; **, *P < *0.01 (based on Student’s *t*-test). (B) The cytotoxicity of RAW264.7 cells after infection with the E. faecalis WT, Δ*pmi*, and ΔPTS1 strains. More than three replicates were performed. (C) The internalization of the E. faecalis WT, Δ*pmi*, and ΔPTS1 strains by RAW264.7 cells at 2 h.p.i. *n *≥ 3; *, *P < *0.05; ***, *P < *0.001 (based on Student’s *t*-test). (D) The *in vitro* growth of the E. faecalis WT strain and the mutant with transposon insertion in OG1RF_12558 to OG1RF_12559, named OG1RF::Tn. Three independent biological replicates were performed. (E) RNA-Seq was performed with RAW264.7 cells infected with E. faecalis WT and ΔPTS1 for 48 h, and KEGG orthology categorization of genes showed significantly different expression in RAW264.7 cells. Download FIG S3, TIF file, 1.9 MB. Copyright © 2021 Wei et al. 2021 Wei et al. https://creativecommons.org/licenses/by/4.0/ This content is distributed under the terms of the Creative Commons Attribution 4.0 International license.

### Carbohydrate metabolism influences E. faecalis survival in macrophages.

The mutants of PTS1 and *pmi* represented carbohydrate metabolism defects or attenuation in E. faecalis and were employed to investigate the function of carbohydrate metabolism during E. faecalis survival of macrophages. Compared with that of the WT strain at 48 or 72 h.p.i., the survival of the Δ*pmi* and ΔPTS1 strains in RAW264.7 cells was significantly enhanced (Fig. 2D). The cytotoxicity of RAW264.7 cells infected by E. faecalis WT, Δ*pmi*, and ΔPTS1 strains exhibited no obvious differences (Fig. S3B), suggesting that the carbohydrate metabolism of E. faecalis has no cytotoxicity to host cells. Moreover, the viable bacterial counts of Δ*pmi* and ΔPTS1 in primary peritoneal macrophages were also significantly greater than that of the WT strain at 48 h.p.i. (Fig. 2E). After the E. faecalis WT, Δ*pmi*, and ΔPTS1 strains were enabled to colonize RAW264.7 cells for 48 h, the survival status of the strains was monitored by TEM, with the results showing that all of the strains were present in phagosomes (Fig. 2F). Notably, more ΔPTS1 than WT bacteria were present in infected cells (Fig. 2F), while no significant increase in the numbers of Δ*pmi* bacteria compared with WT bacteria was observed in infected cells (Fig. 2F), possibly due to the lower internalization of Δ*pmi* than WT (Fig. S3C). Moreover, reverse transcription-quantitative (qRT-PCR) analysis showed that the expression of PTS1 significantly decreased in RAW264.7 cells compared with BHI medium at 24 h of cultivation, and slightly lower expression levels of *pmi* were observed in RAW264.7 cells than in BHI medium (Fig. 2G). These results suggested that E. faecalis downregulates carbohydrate metabolism to promote its own survival in macrophages.

Furthermore, the influence of carbohydrate metabolism on E. faecalis survival in the host was investigated using mice as models. In this experiment, the mutant with a transposon insertion in OG1RF_12558 to OG1RF_12559 (named OG1RF::Tn), which was gentamicin resistant and exhibited no obvious growth defect *in vitro* (Fig. S3D), was used to distinguish between other E. faecalis strains. The OG1RF::Tn strain was mixed 1:1 with the E. faecalis WT, Δ*pmi*, or ΔPTS1 strains and inoculated into the skin of the hind legs of mice at a dose of 10^7^ CFU. Three days postinfection, the Δ*pmi* and ΔPTS1 strains showed significantly lower survival than the WT strain (Fig. 2H), which may have been due to the growth defect of the Δ*pmi* and ΔPTS1 strains observed under *in vitro* conditions (Fig. 2C and Fig. S3A). However, at 6 days postinfection, the Δ*pmi* and ΔPTS1 strains showed, similar to the WT, a competitive index of approximately 1.0, suggesting that the Δ*pmi* and ΔPTS1 strains may resist immune clearance in mice better than the E. faecalis WT strain. To address this possibility, competitive assays of the E. faecalis WT, Δ*pmi*, and ΔPTS1 strains in primary peritoneal macrophages were conducted. The experimental results showed that Δ*pmi* and ΔPTS1 had lower rates of internalization (2 h.p.i.) than OG1RF::Tn but outcompeted OG1RF::Tn at 72 h of infection (Fig. 2I). However, the competitive index of WT to OG1RF::Tn was approximately 1.0 at 2 or 72 h postinfection. The above results suggested that attenuated carbohydrate metabolism facilitated the resistance of E. faecalis to macrophage-mediated killing, enabling longer survival in the host.

### Macrophages respond to carbohydrate metabolism of E. faecalis to activate NO production.

The mechanism by which carbohydrate metabolism in E. faecalis affects macrophage-mediated killing was further explored. As shown in Fig. 2F and in other studies, E. faecalis colonized phagosomes of host cells after internalization (11, 12). In macrophages, the acidification and maturation of phagosomes promote the clearance of pathogens (9, 10). Therefore, we assessed the fitness of E. faecalis strains under low pH, reactive oxygen species (ROS), and lysozyme conditions, which were similar to acidified phagosome conditions. Zou and Shankar previously reported that E. faecalis-containing phagosomes have a pH of 5.82 at 3 h.p.i. (12). Compared with the WT strain, the ΔPTS1 strain exhibited significantly enhanced resistance to different acidic conditions, including pH values of 3.0, 4.0, and 5.0, and the complementation of PTS1 restored the sensitivity of this strain to that of the WT strain (see Fig. S4A in the supplemental material). Δ*pmi* showed slightly higher acidic resistance (pH values of 4.0 to 5.0) than the WT strain, and the *pmi*-complemented strain showed greater sensitivity to acidic conditions (Fig. S4A). In addition, E. faecalis was highly sensitive to pH 3.0 and 4.0 but could survive at pH 5.0 (more than 48 h) (Fig. S4A). Moreover, the deletions of PTS1 and *pmi* did not enhance the resistance of E. faecalis to ROS or lysozyme (data not shown), and infections with these two strains did not clearly attenuate the acidification of phagosomes (Fig. S4B), the levels of ROS (Fig. S4C), or the activities of cathepsin B (Fig. S4D) in RAW264.7 cells compared with WT infection.

10.1128/mSystems.00434-21.4 FIG S4 (A) Sensitivity of the E. faecalis WT, Δ*pmi*, *pmi*^+^, ΔPTS1, and PTS1^+^ strains to acidic conditions, as assessed by inoculating 1.5 × 10^8^ CFU of each strain into modified M9 medium supplemented with 200 μM glucose as the sole carbon resource and adjusting the pH of the medium to 3.0, 4.0, and 5.0. At the indicated time points, the viable bacteria were counted by plating. *n *≥ 3; *, *P < *0.05; ***, *P < *0.001 (based on Student’s *t*-test). (B to D) Flow cytometry analyses of the acidification of phagosomes (B), production of reactive oxygen species (ROS) (C), and activities of cathepsin B (D) after E. faecalis WT, Δ*pmi*, and ΔPTS1 strain infection. The flow cytometry results were analyzed using FlowJo. More than three independent biological replicates were performed, and the MFI and SD are shown. Download FIG S4, TIF file, 2.5 MB. Copyright © 2021 Wei et al. 2021 Wei et al. https://creativecommons.org/licenses/by/4.0/ This content is distributed under the terms of the Creative Commons Attribution 4.0 International license.

**FIG 3:**
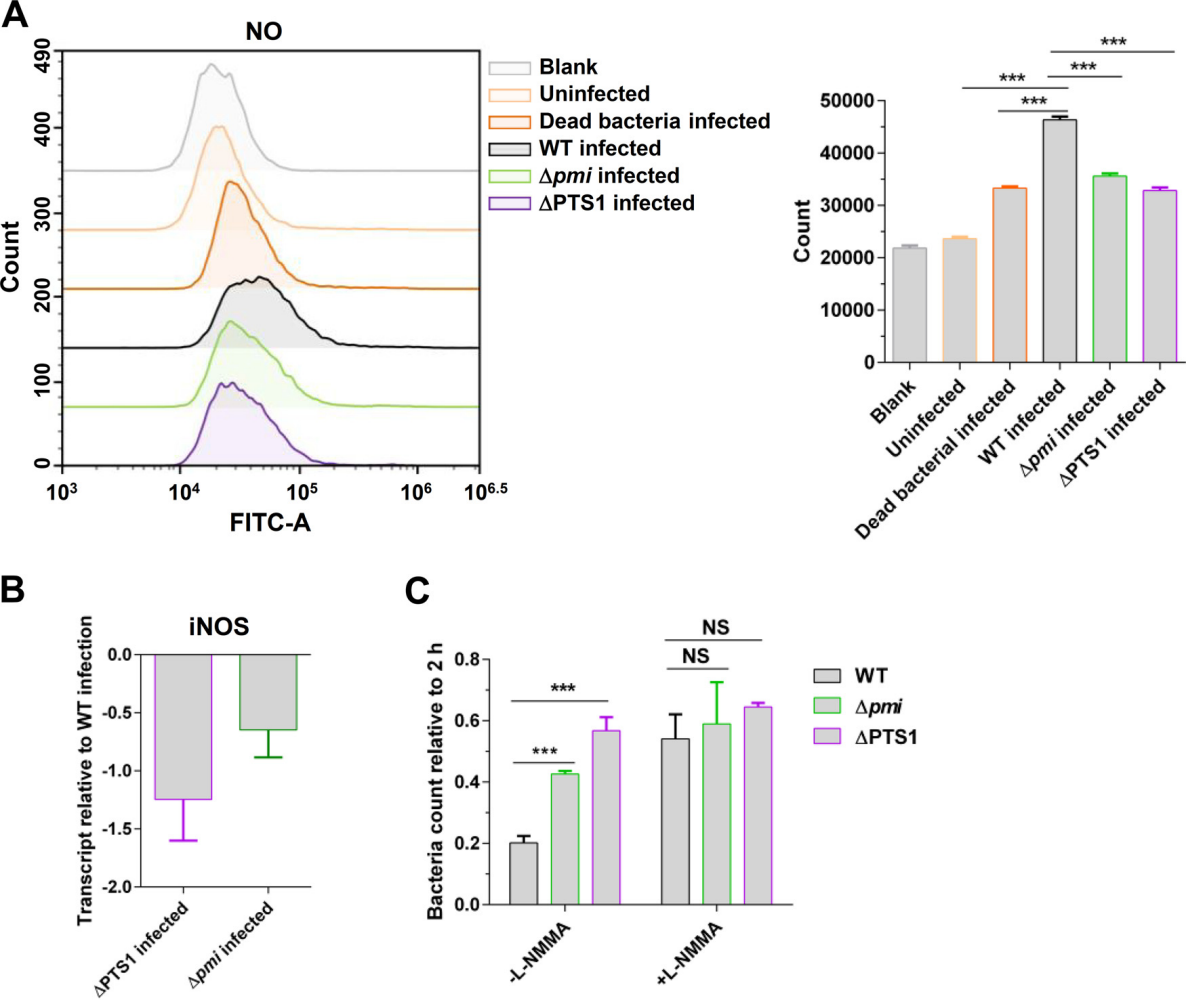
Carbohydrate metabolism of E. faecalis promotes NO production in macrophages. (A) Flow cytometry analyses of the level of NO in RAW264.7 cells after infection with the E. faecalis WT, Δ*pmi*, and ΔPTS1 strains. The dead bacteria represent the E. faecalis WT strain killed by heat. The number represents the mean fluorescence intensity (MFI) with standard deviation (SD). (B) qRT-PCR analysis of the transcription of iNOS in RAW264.7 cells. More than three independent biological replicates were performed. (C) The survival of E. faecalis strains in RAW264.7 cells after 48 h of infection. L-NMMA (300 μM) was added to inhibit the production of NO in RAW264.7 cells. The viable bacterial number at the indicated time was counted by plating. *n *≥ 3; **, *P < *0.01 (based on Student’s *t* test).

Notably, based on the flow cytometry analysis, compared with E. faecalis WT (mean fluorescence intensity [MFI], 46,367) infection, infection with Δ*pmi* (MFI, 35,624) and ΔPTS1 (MFI, 32,851) significantly decreased the accumulation of nitric oxide (NO) in RAW264.7 cells (Fig. 3A). Meanwhile, we assessed the expression of NO synthase (iNOS) in RAW264.7 cells after infection with E. faecalis WT, Δ*pmi*, and ΔPTS1 strains. Compared with infection by the WT strain, infection by ΔPTS1 and Δ*pmi* decreased the expression of iNOS in RAW264.7 cells (Fig. 3B). The inhibition of NO production in RAW264.7 cells significantly increased the intracellular bacterial counts of E. faecalis WT (FC, 2.68) (Fig. 3C). However, the colonizing advantages of ΔPTS1 and Δ*pmi* in RAW264.7 cells were attenuated compared with WT when the inhibitor (L-NMMA) of NO production was added (Fig. 3C). Taken together, the results described above suggest that macrophages would respond to carbohydrate metabolism of E. faecalis to increase the production of NO.

### Carbohydrate metabolism of E. faecalis activates the polarization of macrophages to M1 to induce the production of NO.

**FIG 4:**
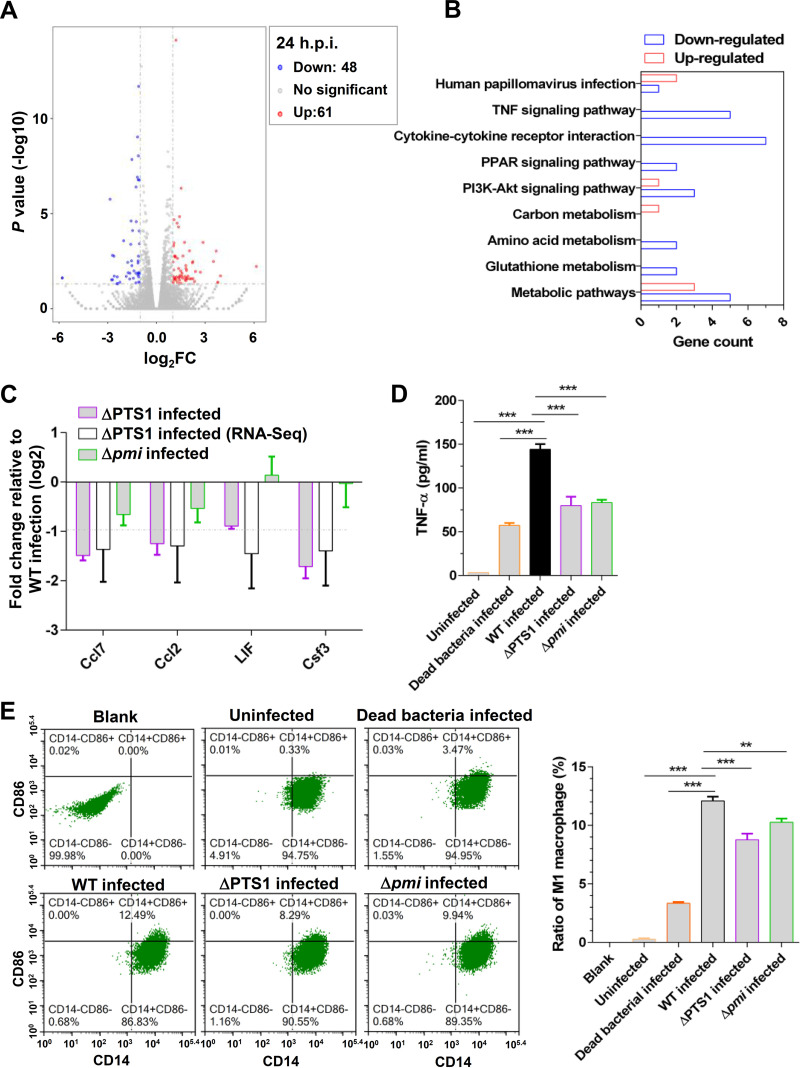
Carbohydrate metabolism in E. faecalis activates the polarization of macrophages to M1 to induce NO production. (A) Volcano plots were utilized to analyze the RNA-Seq results. A total of 61 upregulated genes (red dots) and 48 downregulated genes (blue dots) in RAW264.7 cells infected with the ΔPTS1 strain compared with the WT strain infected at 24 h.p.i. The cutoff was log_2_ FC of ≥1 or ≤−1, and a *P* value of <0.01 was set as significant. Two independent biological replicates were performed. (B) Significantly different genes categorized by KO. (C) Validation of RNA-Seq results by qRT-PCR. More than three independent biological replicates were performed. (D) ELISA analyses of the secretion of TNF-α from RAW264.7 cells. *n *≥ 3; ***, *P < *0.001 (based on Student’s *t* test). (E) Scatterplots were utilized to analyze the polarization of RAW264.7 cells after infection with the E. faecalis WT, Δ*pmi*, and ΔPTS1 strains. RAW264.7 cells were labeled with CD14 and CD86, which is the hallmark of M1 macrophages. Three independent biological replicates were performed, and representative images are shown.

To further investigate the mechanism by which carbohydrate metabolism of E. faecalis activates the production of NO in host cells, transcriptome sequencing (RNA-Seq) was performed with RAW264.7 cells infected by E. faecalis WT and ΔPTS1, which represented defects in carbohydrate metabolism in E. faecalis. The biological replicates of RNA-Seq showed a high correlation (see Table S2 in the supplemental material). Compared with RAW264.7 cells infected by WT E. faecalis, RAW264.7 cells infected by ΔPTS1 elicited the upregulation of 61 genes, and 48 others were downregulated (Fig. 4A). These significantly differentially expressed genes were further analyzed by KEGG orthology, which indicated that the cytokine-cytokine receptor interaction and the tumor necrosis factor (TNF) signaling pathway were clearly upregulated by PTS1 (Fig. 4B). Analysis of qRT-PCR results also showed that PTS1 activated the expression of Ccl7, Ccl2, LIF, and Csf3 (Fig. 4C), which were associated with cytokine-cytokine receptor interactions and the TNF signaling pathway. In addition, infection with Δ*pmi* slightly decreased the expression of Ccl7 and Ccl2 in RAW264.7 cells compared with WT infection (Fig. 4C). Furthermore, the results of enzyme-linked immunosorbent assays (ELISAs) showed that infection with the E. faecalis WT strain notably activated the secretion of TNF-α from RAW264.7 cells (Fig. 4D). However, the secretion of TNF-α from RAW264.7 cells infected by ΔPTS1 and Δ*pmi* was significantly lower than that of cells infected by the WT strain (Fig. 4D). These results were consistent with the RNA-Seq results, suggesting that carbohydrate metabolism in E. faecalis activates inflammatory response in RAW264.7 cells.

10.1128/mSystems.00434-21.6 TABLE S2 RNA-Seq results for RAW264.7 cells infected with the E. faecalis WT and ΔPTS1 strains. Download Table S2, XLS file, 13.2 MB. Copyright © 2021 Wei et al. 2021 Wei et al. https://creativecommons.org/licenses/by/4.0/ This content is distributed under the terms of the Creative Commons Attribution 4.0 International license.

It is well known that iNOS is the hallmark of M1 macrophages (28), and the polarization of macrophages to M1 is involved in inflammatory response and NO production (29). Therefore, we assessed the polarization of RAW264.7 cells after E. faecalis WT, Δ*pmi*, and ΔPTS1 strain infection. An analysis of flow cytometry showed that infection with the E. faecalis WT strain induced the polarization of macrophages to M1 (12.49%) (Fig. 4E), but the heat-killed WT bacteria induced polarization to M1 at 3.47% (Fig. 4E). Moreover, viable bacterial infection also induced more NO and TNF-α production in RAW264.7 cells than heat-killed bacterial infection (Fig. 3B and 4D). These results suggest that viable E. faecalis bacteria strongly induce the polarization of macrophages to M1 to promote inflammation and NO production. Besides, infection with ΔPTS1 (8.29%) and Δ*pmi* (9.94%) showed lower polarization of macrophages to M1 than WT infection (Fig. 4E), suggesting that attenuation of carbohydrate metabolism in E. faecalis decreases the polarization of macrophages to M1 to reduce NO production.

### Carbohydrate shift affects the killing of E. faecalis by macrophages.

**FIG 5:**
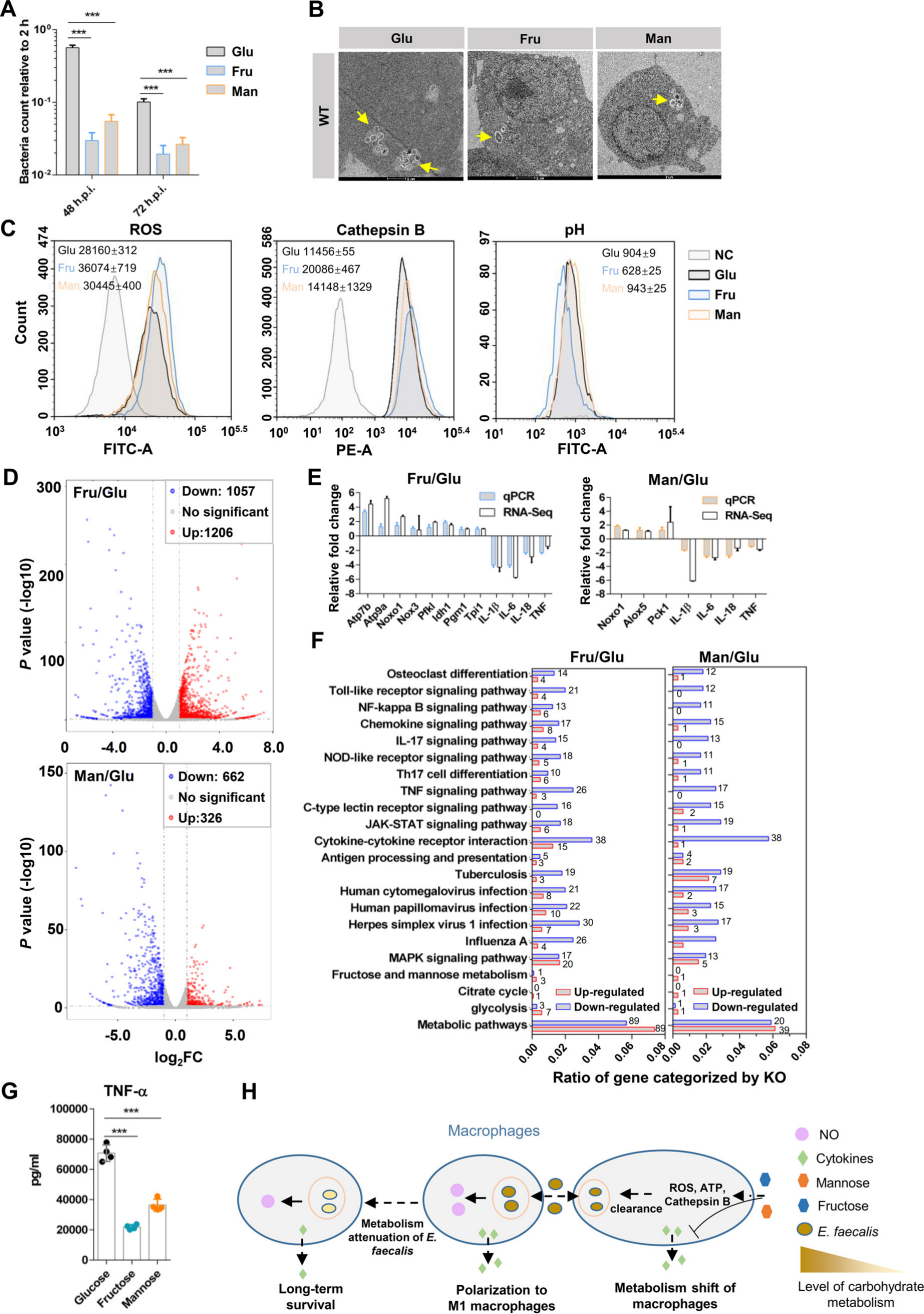
Carbohydrate metabolism affects macrophage-mediated killing of E. faecalis. (A) Sugar shift affects macrophage-mediated killing of E. faecalis. After E. faecalis was internalized by RAW264.7 cells, the extracellular bacteria were killed by gentamicin and vancomycin, and the medium was simultaneously changed to fresh sugar-free DMEM supplemented with glucose, fructose, or mannose as the primary carbon resource. At 48 and 72 h postinfection, the viable bacteria in host cells were counted by plating. *n *≥ 3; ***, *P < *0.001 (based on the Student’s *t* test). (B) TEM analysis showed the status and bacterial number of the E. faecalis WT strain in RAW264.7 cells cultured with sugar-free DMEM supplemented with glucose, fructose, or mannose. Representative images are shown. (C) Flow cytometry analyses of the production of ROS, the activities of cathepsin B, and the acidification of E. faecalis-containing phagosomes in RAW264.7 cells. RAW264.7 cells were treated with different sugars (glucose, fructose, or mannose) and infected with E. faecalis. More than three independent biological replicates were performed. The mean fluorescein intensity (MFI) with standard deviation (SD) was calculated and is shown. (D) Volcano plots were utilized to analyze the RNA-Seq results. RAW264.7 cells treated with glucose, fructose, or mannose were subjected to RNA-Seq. Red dots and blue dots represent upregulation (log_2_FC, ≥1; *P < *0.01) and downregulation (log_2_ FC, ≤−1; *P < *0.01), respectively. Two independent biological replicates were performed. (E) Validation of RNA-Seq results by qRT-PCR. RAW264.7 cells were treated with glucose, fructose, and mannose after E. faecalis infection. The expression of genes in RAW264.7 cells treated with fructose and mannose compared to glucose is shown. Three independent biological replicates were performed. (F) KO categorization of genes with significant differences according to RNA-Seq results. The gene ratio represents the number of genes enriched by one category relative to all the significant genes counted. The number of depleted genes in each category is shown. (G) ELISA analyses of the secretion of TNF-α by RAW264.7 cells treated with glucose, fructose, and mannose after E. faecalis infection. *n *≥ 3; ***, *P < *0.001 (based on Student’s *t* test). (H) Schematic diagram shows carbohydrate metabolism coordinating the survival of E. faecalis in macrophages. Macrophages sense the stimulation of carbohydrate metabolism or metabolites from E. faecalis and polarize to M1 to induce the production of NO for bacterial killing. On the other hand, the shift in carbohydrate metabolism affects macrophage-mediated killing of E. faecalis and inflammation.

The influence of carbohydrate metabolism on macrophage-mediated killing was also investigated. To manipulate the carbohydrate metabolism of RAW264.7 cells, RAW264.7 cells were cultured in sugar-free DMEM supplemented with glucose, fructose, or mannose as the primary carbon resource after internalization of the E. faecalis WT strain. At 48 or 72 h.p.i., the intracellular bacterial counts in the RAW264.7 cells treated with fructose and mannose were significantly lower than in those of cells treated with glucose (Fig. 5A). The survival status of the E. faecalis WT strains in RAW264.7 cells was monitored by TEM, which showed that the addition of exogenous fructose and mannose dramatically decreased the bacterial counts in RAW264.7 cells compared with that observed in glucose-treated cells (Fig. 5B). These results suggested that fructose and mannose enhance macrophage-mediated killing of E. faecalis. To explore the mechanism governing this phenomenon, the production of intracellular ROS, the activities of cathepsin B, and the acidification of E. faecalis-containing phagosomes were detected by flow cytometry. Compared with RAW264.7 cells treated with glucose and E. faecalis infection, fructose and mannose significantly increased the production of ROS and the activities of cathepsin B in RAW264.7 cells after E. faecalis infection (Fig. 5C). In addition, RAW264.7 cells treated with fructose (MFI, 628) exhibited a lower pH in E. faecalis-containing phagosomes than cells treated with glucose and mannose, but no clear difference in pH between glucose (MFI, 904) and mannose (MFI, 934) was observed (Fig. 5C).

To further investigate the influence of carbohydrate on macrophage-mediated killing, RNA-Seq was performed (see Table S3 and S4 in the supplemental material). According to the RNA-Seq results, fructose significantly increased the expression levels of 1,206 genes and decreased the expression levels of 1,057 genes (Fig. 5D and Table S3), and mannose significantly increased the expression levels of 326 genes and decreased the expression levels of 662 genes compared with those in RAW264.7 cells treated with glucose and E. faecalis infection (Fig. 5D and Table S4). Among these significantly different genes, ROS-related genes in RAW264.7 cells treated with fructose (Noxo1 log_2_FC, 2.69; Nox3 log_2_FC, 1.01) and mannose (Noxo1 log_2_FC, 1.28; Alox5 log_2_FC, 1.15; Alox12 log_2_FC, 1.44) were significantly upregulated compared with glucose-treated cells (Table S3 and S4). ATP synthase-related genes, which promote the acidification of E. faecalis-containing phagosomes, showed significant increases in RAW264.7 cells treated with fructose (Atp7b log_2_FC, 5.14; Atp9a log_2_FC, 5.18; Atp6v0d2 log_2_FC, 2.74) (Table S3). However, the cathepsin B gene (Ctsb) showed no significant change in fructose (log_2_FC, 0.46) and mannose (log_2_FC, −0.45) compared with glucose (Table S3 and S4). qRT-PCR analysis was performed to validate the RNA-Seq results and showed that fructose significantly increased the expression of ROS-related genes (Noxo1 and Nox3) and ATP synthesis-related genes (Atp7b and Atp9a) in RAW264.7 cells compared with glucose (Fig. 5E), which was consistent with the RNA-Seq results. In addition, mannose significantly increased the expression levels of Noxo1 and Alox5, of which both promoted the production of ROS in RAW264.7 cells.

10.1128/mSystems.00434-21.7 TABLE S3 RNA-Seq for RAW264.7 cells treated with glucose and fructose. Download Table S3, XLS file, 13.1 MB. Copyright © 2021 Wei et al. 2021 Wei et al. https://creativecommons.org/licenses/by/4.0/ This content is distributed under the terms of the Creative Commons Attribution 4.0 International license.

10.1128/mSystems.00434-21.8 TABLE S4 RNA-Seq for RAW264.7 cells treated with glucose and mannose. Download Table S4, XLS file, 13.1 MB. Copyright © 2021 Wei et al. 2021 Wei et al. https://creativecommons.org/licenses/by/4.0/ This content is distributed under the terms of the Creative Commons Attribution 4.0 International license.

According to the analysis of RNA-Seq results based on KEGG orthology, fructose and mannose significantly upregulated energy metabolism, such as fructose and mannose metabolism, citrate cycle, glycolysis, and other metabolic pathways, compared with RAW264.7 cells treated with glucose and infected with E. faecalis (Fig. 5E and F). In addition, fructose and mannose dramatically downregulated the expression levels of innate immunity components, including the Toll-like receptor signaling pathway, NF-κB signaling pathway, chemokine signaling pathway, interleukin-17 (IL-17) signaling pathway, and TNF signaling pathway (Fig. 5E to G), which implies that fructose and mannose promote macrophage-mediated killing independent of inflammatory responses. The results described above showed that carbohydrate metabolism affects the ability of macrophages to mediate killing of E. faecalis and simultaneously represses the activation of inflammation compared with glucose treatment.

## DISCUSSION

E. faecalis resists immune clearance by the host and causes severe infectious diseases worldwide. Although several factors have been reported to contribute to the resistance mechanisms of E. faecalis against host immunity, such as the enterococcal polysaccharide antigen (EPA) (30), the impact of metabolites or metabolic pathways on the ability of E. faecalis to resist macrophage-mediated killing has rarely been reported. According to the results of transposon insertion sequencing, a total of 15 and 19 mutants with decreased fitness in RAW264.7 cells were identified from the 48- and 72-h groups, respectively, of which 9 overlapped (Fig. 1D). Among these nine mutants, a mutation in *rpoN*, which was screened out for causing a fitness reduction (log_2_FC, −2.18) (Table S1), has been reported to promote the killing of phagocytes by Pseudomonas aeruginosa (31). The other genes with disruptions that caused fitness reductions included those encoding an acetyltransferase (log_2_FC, −1.66), aspartate aminotransferase (log_2_FC, −1.59), and a LysR family transcriptional regulator (log_2_FC, −1.73) (Table S1), of which all will be further investigated in the future. In addition, mutants in OG1RF_11714, which encodes a polysaccharide antigen EPA-related protein, glycosyltransferase, displayed an abundance reduction after 48 h (log_2_FC, −0.713) or 72 h (log_2_FC, −0.829) of survival in RAW264.7 cells (Table S1), which was consistent with the results reported by Smith (30).

Bacteria depend on the phosphotransferase system (PTS) to sense and transport extracellular carbohydrates. The PTS has been also reported to affect virulence gene expression and infection in Streptococcus sp., Bacillus anthracis, and Vibrio cholerae (32–34), and mannose metabolites can also act as signaling molecules to coordinate virulence gene expression and influence infection (25). Surface components, including teichoic acids, a capsule, and enterococcal polysaccharide antigen, play key roles for *Enterococcus* sp. escape from phagocytosis (35–37). In addition to affecting cellular resistance to low pH (Fig. 4A), carbohydrate metabolism may also mediate virulence factor expression or surface components to affect E. faecalis resistance to macrophage-mediated killing, which warrants further investigation.

Metabolic interactions are important in the battle between pathogens and hosts (38–40). When sensing bacterial invasion, the host reprograms metabolism to aerobic glycolysis, which is called the Warburg effect (41). The metabolism shift leads to the accumulation of succinate, which induces inflammation (42). Therefore, some bacterial pathogens need to coordinate their own metabolism to escape from immune activation of the host. M. tuberculosis enters a dormant-like status after colonizing host cells, enhancing their ability to resist immune clearance or avoid immune activation (43). Our results demonstrate that E. faecalis attenuates carbohydrate metabolism to escape from the activation of host immunity and facilitates its own survival in macrophages (Fig. 5H). Fan et al. also showed that the gluconate metabolism of E. faecalis activates the inflammation of macrophages (44). However, how carbohydrate metabolism of E. faecalis triggers immune activation has not been investigated to date. To figure out this question, the transcriptomes of RAW264.7 cells infected with the E. faecalis WT and ΔPTS1 strains were performed (48 h.p.i.) and showed that PTS1 affected the energy metabolism of RAW264.7 cells, including the insulin signaling pathway, pyruvate metabolism, fructose and mannose metabolism, pentose phosphate pathway, citrate cycle, insulin resistance, phosphatidylinositol 3-kinase (PI3K)-Akt signaling pathway, and AMP-activated protein kinase (AMPK) signaling pathway (Fig. S3E). Some investigations have also shown that the enhancement of glycolysis and pentose phosphate pathway activity in macrophages promotes rapid energy production and the polarization of macrophages to M1, thereby leading to rapid antibacterial and inflammatory responses (45). Thus, the metabolites of E. faecalis may trigger the host to reprogram its own metabolism for the immune response.

Compared with glucose, fructose and mannose (especially fructose) have been shown promoting the macrophage-mediated killing by increasing the production of ROS, the activities of cathepsin B, and the acidification of phagosomes (Fig. 5C), which is independent of inflammatory response (Fig. 5E and F). Thus, we speculate that the shift of metabolism may affect nutrition obtained by E. faecalis in macrophages. According to TEM images (Fig. 2F, 5B, and S1B), some E. faecalis bacteria were dividing in host cells after 24 or 48 h of survival, implying that E. faecalis may replicate in macrophages. The shift of carbohydrates for macrophage cultivation may affect the nutrient acquisition of E. faecalis during intracellular survival. Xian et al. reported that Ralstonia solanacearum hijacks host metabolites to support its own replication (38). Thus, whether E. faecalis hijacks host carbohydrates as nutrients to promote survival warrants further study.

In summary, the results of the present study helped to elucidate the mechanism by which carbohydrate metabolism mediates the macrophage-mediated killing of E. faecalis (Fig. 5H), which may provide important strategies for the treatment of chronic inflammatory and infectious diseases caused by E. faecalis.

## MATERIALS AND METHODS

### Bacterial strains, plasmids, and growth conditions.

Bacterial strains and plasmids used in this study are described in Table S5 and oligonucleotides in Table S6 in the supplemental material. E. faecalis strains were routinely grown at 37°C or 42°C (inducing transposition) in brain heart infusion (BHI) broth or agar. E. coli and L. lactis were grown in Luria-Bertani (LB) broth or agar, and erythromycin (Erm) was added at a final concentration of 200 μg/ml to select pTetH or pGhost derivatives. For complementation assays, erythromycin was used at a final concentration of 30 μg/ml to select positive colonies and anhydrotetracycline (Tet) was used at a final concentration of 10 ng/ml to induce gene expression.

10.1128/mSystems.00434-21.9 TABLE S5 Bacterial strains and plasmids used in this study. Download Table S5, XLS file, 0.03 MB. Copyright © 2021 Wei et al. 2021 Wei et al. https://creativecommons.org/licenses/by/4.0/ This content is distributed under the terms of the Creative Commons Attribution 4.0 International license.

10.1128/mSystems.00434-21.10 TABLE S6 Primers used in this study. Download Table S6, XLS file, 0.02 MB. Copyright © 2021 Wei et al. 2021 Wei et al. https://creativecommons.org/licenses/by/4.0/ This content is distributed under the terms of the Creative Commons Attribution 4.0 International license.

### Transposon insertion sequencing and mapping.

The construction of the transposon insertion library of E. faecalis was described by Wei et al. (46). High-throughput sequencing and analysis were previously described (21, 47). Briefly, genomic DNA was extracted and fragmented by sonication, followed by end repairing, A-tailing, and addition of adaptors and P5/P7 sequences by PCR. Three or two library replicates of inputs and outputs were applied to high-throughput sequencing on the Illumina HiSeq 2500 platform. The sequencing results were processed with adapter trimming, mapping to the genome, and tallying as previously described (21). The read counts for each locus were normalized among the libraries according to sequencing depth. The fold change of each locus was generated with the output compared with the input read counts. Conditional essential genes were determined using the HMM module of EL-ARTIST (23). Functional classification is based on the KEGG (https://www.genome.jp/kegg/).

### Construction of deletion and complementation.

The in-frame deletion of E. faecalis OG1RF was constructed by allele exchange using the procedure previously described (30). pGhost9 derivatives, which were replicated in a temperature-dependent manner in host cells, were electroporated, and transformants were selected at 28°C on BHI agar plates with erythromycin (30 μg/ml). Then, E. faecalis harboring the pGhost9 derivative was grown at a nonpermissive temperature (42°C) in the presence of erythromycin to induce single crossover recombination. The second recombination event happened after 7 serial subcultures at 28°C without erythromycin, and the double crossover mutation was identified by PCR and sequencing.

For complementation, DNA fragments coding PTS1 and *pmi* were amplified by PCR using the oligonucleotides described in Table S6. PCR products were ligated to pTetH, which were digested by NcoI and BamHI in advance. The constructed pTetH derivatives were transferred in E. faecalis cells by electroporation, and positive colonies were screened by erythromycin (30 μg/ml) and sequenced. For complementation assays, pTetH derivatives were anhydrotetracycline-inducible expression.

### Infection of macrophages and the intracellular bacterial counting.

The RAW264.7 cells and primary peritoneal macrophages were used as the cell model, and the cell culture and infection procedures were followed as described by Zou (12). Briefly, cells were cultured in Dulbecco’s modified Eagle’s medium (DMEM) supplemented with 10% fetal bovine serum (FBS) at 37°C in 5% CO_2_. A total of 2.0 × 10^5^ cells were seeded into 24-well plates and then infected by E. faecalis strains at a multiple of infection (MOI) of 10 or 20, following immediately by centrifugation at 300 × *g* for 10 min to facilitate bacterial sinking on RAW264.7 cells. At 2 hour postinfection, the extracellular bacteria were then removed by washing with sterile phosphate-buffered saline (PBS), followed addition of vancomycin (16 μg/ml) and gentamicin (150 μg/ml) to kill the extracellular bacteria. Fresh culture medium was changed every 24 h. At the indicated time points, the macrophages were lysed by 4°C 0.1% Triton X-100. The intracellular bacteria were quantified by serial dilution and plate counting. NO inhibitor (L-NMMA) was added if necessary.

### Transmission electron microscopy.

A total of 1 × 10^6^ RAW264.7 cells were seeded into 6-well plates to grow overnight and then infected with E. faecalis strains, E. coli, or L. lactis at an MOI of 20 for 2 h at 37°C. The extracellular bacteria were washed twice with sterile PBS and killed by vancomycin and gentamicin after 2 h postinfection. At the indicated time points, 5% glutaraldehyde was added to fix infected RAW264.7 cells. The fixed cells were dehydrated and embedded in Epon/Araldite, cured, and sectioned. The sections were placed on glow-discharged 150-mesh Cu grids, which were imaged on a Talos L120C transmission electron microscope (TEM). A total of 11 infected cells were random picked up to statistically calculate the average count of intracellular bacteria.

### E. faecalis adaption to acidic conditions.

To test the sensitivity of E. faecalis strains to acidic conditions, the M9 medium supplemented with 200 μM glucose as the sole carbon resource was adjusted to pH 3.0, 4.0, and 5.0. Thus, E. faecalis was capable of surviving into the modified M9 medium but could not grow. E. faecalis strains were inoculated into this modified M9 medium (pH 3.0, 4.0, and 5.0) with a dose of 1.5 × 10^8^ CFU. At the setting time points, the viable bacteria in the modified M9 medium were counted by a series of dilutions and plating onto BHI agar plates.

### Detection of intraphagosomal acidification.

The detection of phagosome acidification was conducted as described previously (48). First, E. faecalis strains were labeled with fluorescein isothiocyanate (FITC; pH sensitive; Thermo Fisher) and Alexa Fluor 647 (pH insensitive; Thermo Fisher) fluorescent dyes. One milliliter of a E. faecalis suspension was mixed with 25-μl FITC and 25-μl Alexa Fluor 647 dye solutions, and the suspension was incubated in the dark for 1 h at room temperature with continuous shaking. The RAW264.7 cells were infected with E. faecalis strains labeled with FITC and Alexa Fluor 647 for 2 h. The extracellular bacteria were washed three times with sterile PBS, and the cells were continually incubated to the indicated time point. The mean fluorescent intensity of FITC and Alexa Fluor 647 was analyzed with flow cytometry.

### Detection of ROS and NO productions and cathepsin B activities.

A total of 4 × 10^5^ RAW264.7 cells were seeded onto 12-well plates and cultured overnight. At 2 h postinfection, RAW264.7 cells were washed three times with PBS, received fresh DMEM medium with 10% FBS, and continued incubating at 37°C for 24 h. At the setting time points, the cells were washed twice with PBS and stained with the MitoSOX Red mitochondrial superoxide indicator (Thermo Fisher) for ROS detection, Magic Red cathepsin B (Bio-Rad) for cathepsin B detection, or 4-amino-5-methylamino-2′,7′-difluorofluorescein diacetate (DAF-FM DA; Beyotime Biotechnology) for NO detection. After being washed three times with PBS, the cells were subjected to analysis by flow cytometry. The mean fluorescent intensity was calculated.

### Total RNA extraction and qRT-PCR.

The total RNA of RAW264.7 cells or E. faecalis was extracted with a commercial RNA isolation kit (BioFlux). The mRNA was reverse transcribed into cDNA by the PrimeScript RT reagent kit (TaKaRa). The qRT-PCR was performed with a LightCycler 480 II instrument (Roche), and the *C_T_* values were analyzed. The relative fold change was calculated with the housekeeping gene *gyrB* (for E. faecalis) or the gene Gapdh (for RAW264.7 cells) as an internal control.

### RNA-Seq and data analysis.

RNA-Seq and data (NCBI accession number PRJNA734659) analysis were conducted as previously described (49). Briefly, total RNA of RAW264.7 cells infected with E. faecalis were extracted with a commercial RNA isolation kit (BioFlux), and mRNA was enriched by magnetic beads. For RNA-Seq, the VAHTS stranded mRNA-Seq library prep kit (Vazyme) was used to construct strand-specific RNA-Seq libraries. After libraries were qualified, the sequencing was conducted on the HiSeq 2500 platform. The Illumina HiSeq sequencer captured the fluorescence signal and converted the optical signal into a sequencing peak through computer software to obtain sequence information on the tested fragments. Kyoto Encyclopedia of Genes and Genomes (KEGG) pathway analysis was carried out based on RNA-Seq data online (https://www.genome.jp/kegg/tool/map_pathway2.html).

### Isolation of primary peritoneal macrophages.

The isolation of primary peritoneal macrophages was performed as previously described (50). Briefly, male C57BL/6 mice (5 weeks old) were each injected with 2 ml aged 3% thioglycolate to induce the production of macrophages. Five days after induction, mice were sacrificed, and primary peritoneal macrophages were isolated from mouse abdomens. After being washed three times with PBS, primary peritoneal macrophages were seeded into 24-well plates and cultured in DMEM supplemented with 10% FBS.

### Mouse infection and competitive index detection.

The competitive index of E. faecalis strains in mice was performed as described by Chong (51). Male C57BL/6 mice (6 to 7 weeks old; 20 to 23 g) were anesthetized with isoflurane and hind leg hair removed. The skin was disinfected with 75% ethanol, and then 5 μl of mix bacteria (1.0 × 10^7^ CFU) were inoculated under the skin. At the indicated time points, the mice were euthanized and a 1 by 1-cm piece of skin surrounding the inoculated site was excised into sterile PBS. The excised skins were homogenized, and viable bacteria were counted by diluted plating on BHI agar supplemented with antibiotics. The strain with an intergenic insertion of the transposon between OG1RF_12558 and OG1RF_12559 (named OG1RF::Tn) was used to distinguish Δ*pmi* and ΔPTS1 according to gentamicin resistance.

### Ethics statement.

All the procedures mentioned in this study were approved by the Institutional Ethical Review Board of Shanghai Ninth People’s Hospital, School of Medicine, Shanghai Jiao Tong University, and conducted in conformity with institutional guidelines for the care and use of laboratory animals in School of Medicine, Shanghai Jiao Tong University.

### Statistical analyses.

Statistical analyses were performed using GraphPad Prism version 6.0. The results were representative of at least three independent experiments (*, *P < *0.05; **, *P < *0.01; ***; *P < *0.001 [Student’s *t* test or ANOVA]).

### Data availability.

Raw sequencing data in FASTQ format are publicly available for download through the Data Repository for NCBI at https://www.ncbi.nlm.nih.gov/sra/PRJNA734659.
