# Supplementary figures and images for: Carbohydrate Metabolism Affects Macrophage-Mediated Killing of Enterococcus faecalis
Source: mSystems. 2021 Sep 7;6(5):e00434-21. doi: 10.1128/mSystems.00434-21 (PMC8547443; doi:10.1128/mSystems.00434-21)

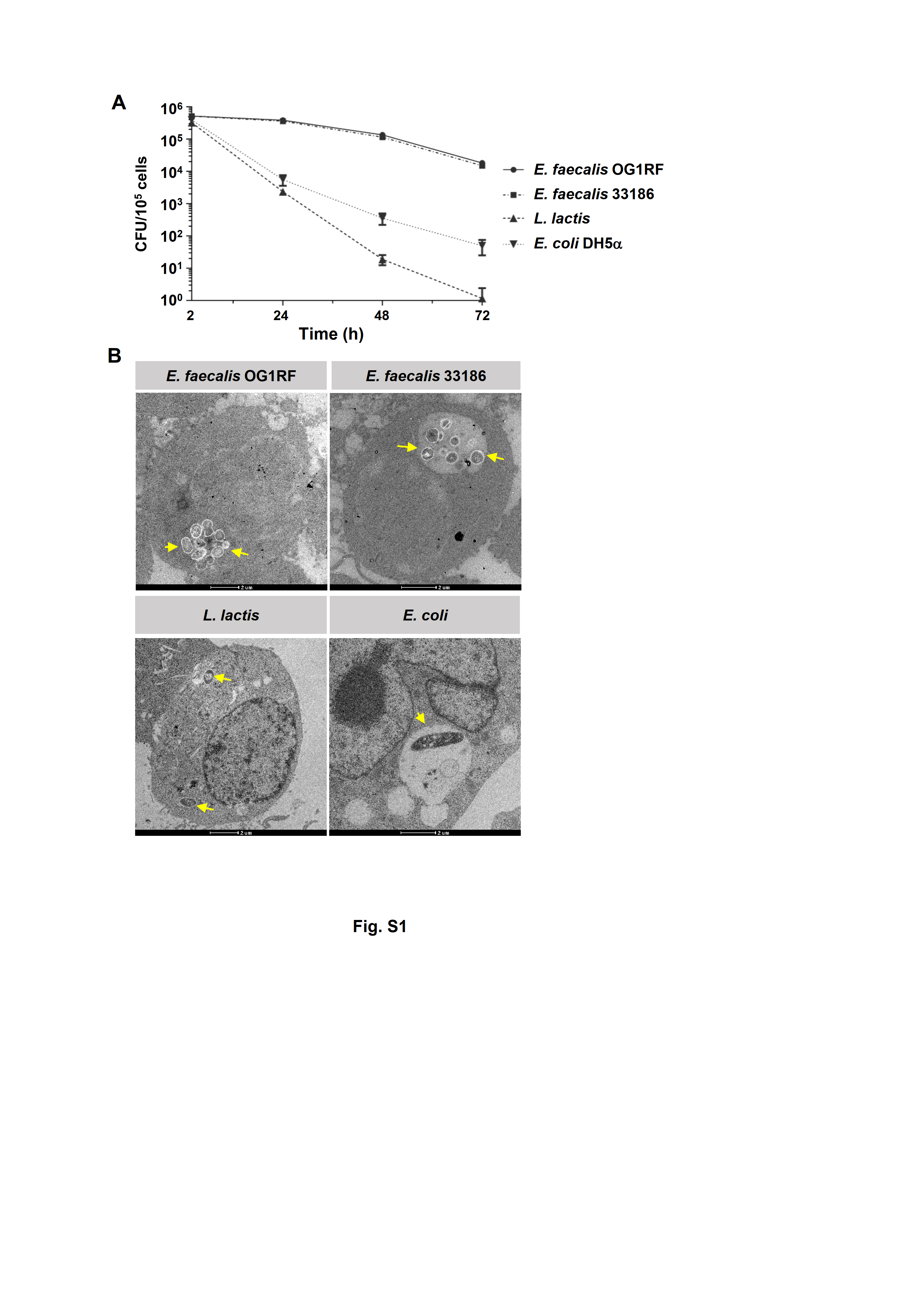

Supplement: FIG S1 [file msystems.00434-21-sf001.tif]

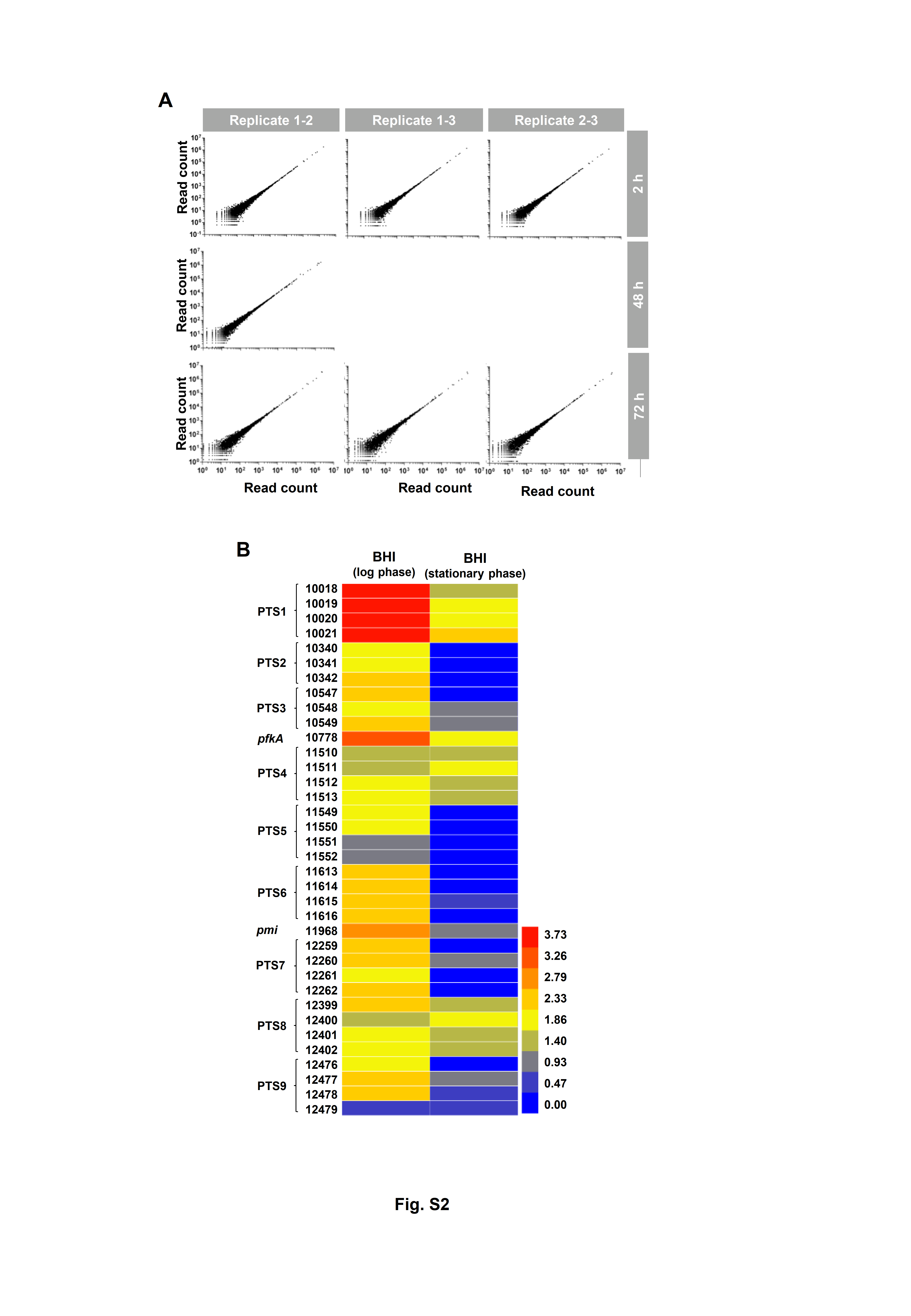

Supplement: FIG S2 [file msystems.00434-21-sf002.tif]

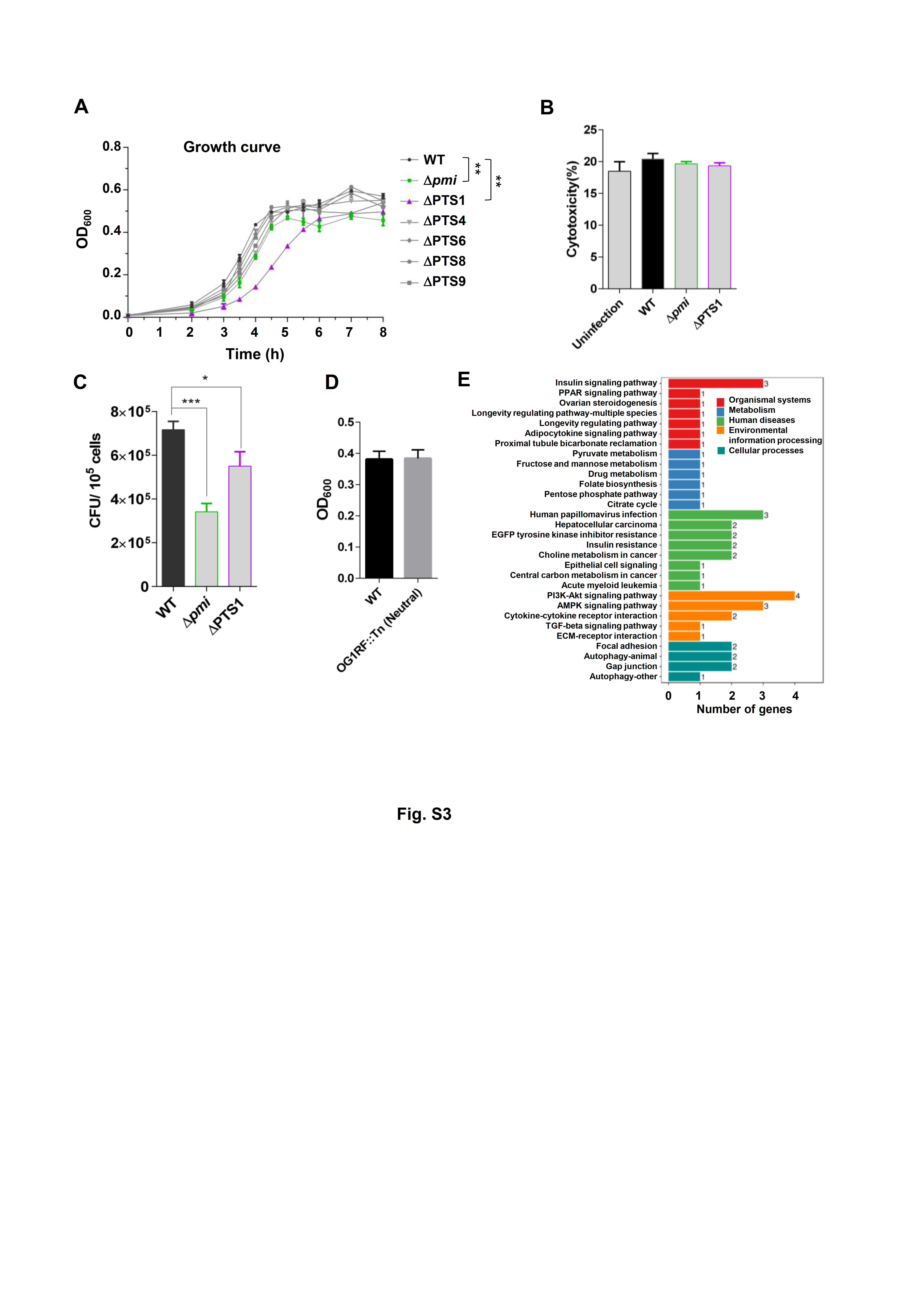

Supplement: FIG S3 [file msystems.00434-21-sf003.tif]

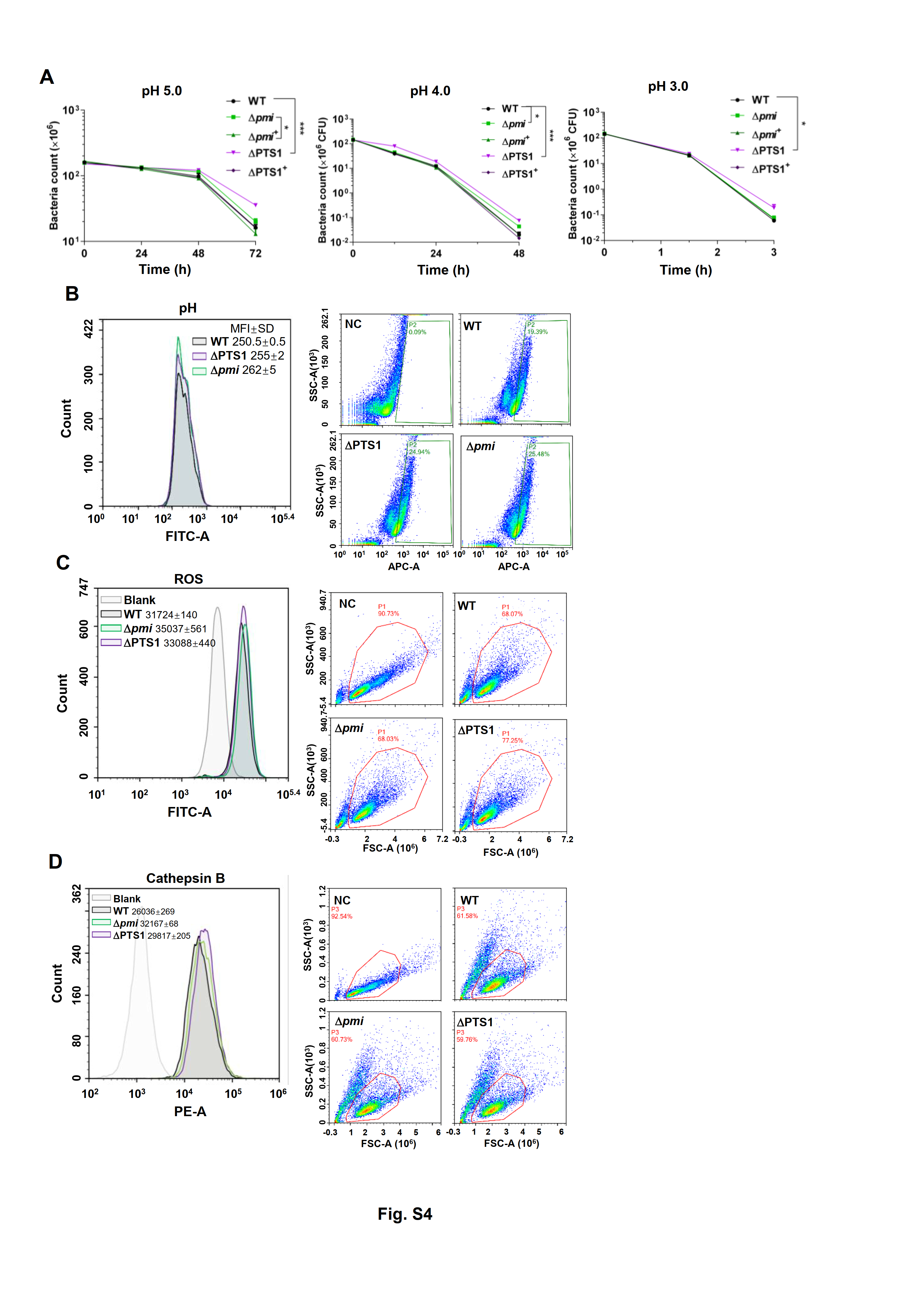

Supplement: FIG S4 [file msystems.00434-21-sf004.tif]
